# Supplementary material for: Few regulatory metabolites coordinate expression of central metabolic genes in Escherichia coli
Source: Mol Syst Biol. 2017 Jan 3;13(1):903. doi: 10.15252/msb.20167402 (PMC5293157; doi:10.15252/msb.20167402)
Supplement: Supplementary file 1 — Appendix [file MSB-13-903-s001.pdf]

Appendix for

Few regulatory metabolites coordinate expression of central metabolic genes in *Escherichia coli*

Karl Kochanowski*, Luca Gerosa*, Simon Felix Brunner, Dimitris Christodoulou, Yaroslav V. Nikolaev, Uwe Sauer

*: equal contribution

Contents

1 Appendix 2
2 1. Mathematical model of promoter activity 2
3 2. Using correlation to infer promoter-metabolite interactions from steady-state data 3
4 3. Algorithm to systematically identify regulatory metabolites 6
5 Appendix figures 7
6 EV tables 23
7 References 24

Appendix

1. Mathematical model of promoter activity

In this section, we develop a description of gene expression regulation by two mechanisms: global regulation by the expression machinery and specific regulation by metabolite-binding transcription factors. The model accounts for the contributions of the expression machinery and of transcription factors representing global and specific regulation, respectively (**eq. 1 in the main text**):

$$\mathrm{pa_{ij}} = \left(\mathrm{E_j}/\mathrm{K_{Ei}}\right)^{\alpha_{\mathrm{Ei}}} \cdot \prod_{\mathrm{l} \in \{\mathrm{TF}\}} \left(1 + \mathrm{TF_{lj}}\ /\mathrm{K_{li}}\right)^{\alpha_{\mathrm{li}}}$$

Here, $\mathrm{pa_{ij}}$ denotes the activity of promoter i in condition j, E denotes the activity of the expression machinery (in condition j) and TF denotes the activity of each specific transcription factor l (in condition j). The two parameters K and α associated to each activity represent biochemical affinities and cooperative or saturating mechanisms (approximated by power law terms for mathematical convenience, see below), respectively. In this representation, transcription factors can act as activators ($\alpha_{li} > 0$) or inhibitors ($\alpha_{li} < 0$). The activity of each transcription factor l can in turn be described as follows (**eq. S1**):

$$\mathrm{TF_{lj}} = \mathrm{TF_{conc\,lj}} \prod_{\mathrm{k} \in \{\mathrm{M}\}} \left(1 + \mathrm{M_{kj}}/\mathrm{K_{lk}}\right)^{\beta_{\mathrm{lk}}}$$

where $\mathrm{TF_{conc\ lj}}$ denotes the transcription factor concentration (in condition j), M denotes the concentration of each metabolite acting as a regulator of transcription factor activity with its transcription factor specific parameters K_{lk} and β_{lk}. Metabolites can activate ($\beta_{lk} > 0$) or inhibit ($\beta_{lk} > 0$) transcription factor activity.

The relationship between promoter activity and global/specific regulation described in equations 1 and S1 can be simplified by first transforming them into log space, second by approximating each term expressed as log(1+x) with log(x), and third by normalizing e.g. for a reference condition to eliminate unknown kinetic parameters ($\Delta \log(x_i) = \log(x_i) - \log(x_{ref})$)(**eq. S2**):

$$\Delta\log\left(\mathrm{pa_{ij}}\right) \approx \alpha_{\mathrm{Ei}} \cdot \Delta\log\left(\mathrm{E_j}\right) + \sum_{\mathrm{l} \in \{\mathrm{TF}\}} \alpha_{\mathrm{li}} \cdot \Delta\log\left(\mathrm{TF_{conc\,lj}}\right) + \sum_{\mathrm{l} \in \{\mathrm{TF}\}, \mathrm{k} \in \{\mathrm{M}\}} \alpha_{\mathrm{li}} \cdot \beta_{\mathrm{lk}} \cdot \Delta\log\left(\mathrm{M_{kj}}\right)$$

We can further simplify equation S2 by assuming that transcription factor expression does not change significantly across conditions, as has been shown for *E. coli* (Ishihama *et al*, 2014; Gerosa *et al*, 2015) (**eq. S3**):

$$\Delta\log\left(\mathrm{pa_{ij}}\right) \approx \alpha_{\mathrm{Ei}} \cdot \Delta\log\left(\mathrm{E_j}\right) + \sum_{\mathrm{l} \in \{\mathrm{TF}\}, \mathrm{k} \in \{\mathrm{M}\}} \alpha_{\mathrm{li}} \cdot \beta_{\mathrm{lk}} \cdot \Delta\log\left(\mathrm{M_{kj}}\right)$$

Thus, in this linearized approximation promoter activity can be decomposed into linear contributions from the expression machinery activity (= global regulation) and the concentration of the metabolites which regulate the activity of the respective transcription factors (= specific regulation).

2. Using correlation to infer promoter-metabolite interactions from steady-state data

While equation S3 describes the general case of a promoter under both global regulation and specific regulation by multiple transcription factors with multiple metabolite effectors, regulatory circuits operating in *E. coli* metabolism are likely to be simpler (i.e. with fewer relevant regulatory inputs). In the following section, we assess if and when operating regulatory circuits are in theory identifiable by correlating steady state measurements of promoter activity and metabolite concentrations.

There are many factors that can confound correlations of promoter activity and metabolites within regulatory circuits, as well as factors that can lead to correlations even without direct regulatory mechanistic links. Factors that can confound true regulatory links are measurement noise, ignorance of kinetic parameters (or more complex interaction mechanisms for which the model approximation above does not apply), and combinatorial contributions of many regulators and metabolites. Factors that can lead to correlations without direct links are cross-correlations between metabolites or transcription factors in many conditions, e.g. due to co-regulation. Given these limitations, how and when can metabolite-gene links mediated by transcription factors being identified by correlations?

To address these issues, we performed simulations assuming single-input promoters (= specific regulation is determined by the concentration of a single metabolite) and using the same number of data points per promoter (n=23 conditions for which we quantified metabolite concentrations and promoter activities) as in our experimental data (see simulation steps at the end of the section).

In particular, we focused on two aspects: first, when is it necessary to remove the confounding effect of global regulation to ensure the recovery of potential regulatory metabolites? To address this question, we grouped the simulated promoter activities based on the relative contribution of the expression machinery (determined as the Pearson correlation coefficient between log transformed promoter activity and expression machinery activity, small contribution: $R < 0.25$, moderate contribution: $0.25 < R < 0.75$, large contribution: $R > 0.75$) and calculated the distribution of correlation coefficients between log transformed metabolite and log transformed promoter activity with or without removing the expression machinery contribution. An interaction is considered to be recovered if the respective correlation coefficient exceeds the threshold (below -0.75, or above 0.75). As expected, for promoters that are predominantly affected by global regulation, the recovery of promoter-metabolite interactions is poor (simulation figure A, right panel). Conversely, for promoters whose activity is dominated by specific regulation, correcting for expression machinery effects has little impact on the recovery of regulatory metabolites (simulation figure A, left panel). These results are consistent with the experimental findings discussed in the main text (figure 4D) and confirm that removing the contribution of global regulation is pivotal especially for promoters whose activity is modulated, but not dominated, by specific regulation.

Second, we wanted to assess the impact of confounding cross-correlating metabolites on our ability to identify true regulatory metabolites. Towards this end, we simulated additional metabolites with 25%, 50%, 75%, 95% similarity to the original simulated metabolite concentration (using Pearson correlation as a similarity metric), which serve as false positives, and determined false discovery and true positive rates (simulation figure B). As illustrated by the resulting ROC curves, confounding metabolites that correlate poorly with the original metabolite (25% or 50% similarity) have a negligible effect on false discovery rates. Even when considering confounding metabolites with higher similarity (75%), the false discovery rate is below 10% when imposing a true positive rate of 75% (meaning that 75% of the true promoter-metabolite interactions present in the data set will be recovered). In the simulations, this true positive rate corresponds to a correlation threshold of 0.74, which justifies the correlation threshold used for the experimental data ($R>0.75$).

Protocol used to simulate data and evaluate the performance of the approximated model in recovering true metabolite-promoter interactions (repeated for 10000 data points):

1. Uniformly sample 23 values in the range [0, 10] for the expression machinery activity $\mathrm{E}^*/\mathrm{K_E}$
2. Uniformly sample 23 values in the range [0, 10] for $[M]/K_M$ (metabolite concentration)
3. Uniformly sample $[T]/K_T$ (transcription factor concentration) in the range [0, 10], add random variation in transcription factor concentration of 15% (sampled from uniform distribution) across conditions
4. Uniformly sample parameters α_{EM} in the range [0, 1.5] and α and β in the range [-4,4]
5. Calculate promoter activity based on sampled expression machinery activity, metabolite and transcription factor concentration, and parameters using **equations 1** and **S1**
6. Add 15% noise sampled from a normal distribution to metabolites and promoter activity data to simulate measurement noise
7. Determine the correlation between log transformed promoter activity (either with or without subtracting the log transformed expression machinery activity) and log transformed metabolite concentration to identify putative promoter-metabolite interactions.
8. For each simulation, generate a metabolite data set with 25%, 50%, 75% similarity (= correlation) to the original metabolite concentration, and repeat step 7.

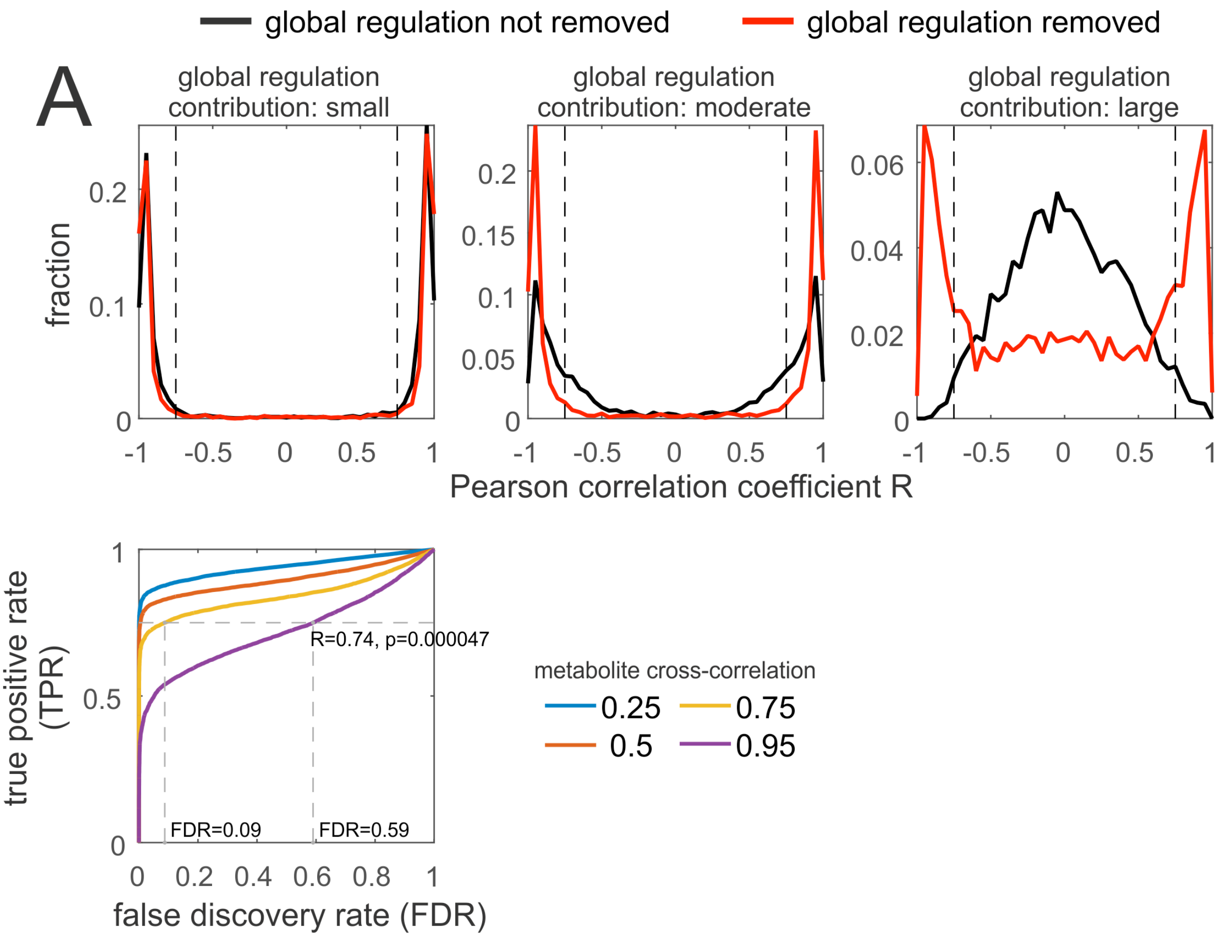

Summary figure of simulations. A) Distribution of Pearson correlation coefficients between simulated metabolite concentration and promoter activity with (red) or without (black) removing the contribution of expression machinery. For each simulated promoter (n = 10000), its expression

machinery contribution was determined as the Pearson correlation coefficient between log-transformed promoter activity and expression machinery activity, and promoters were then grouped into small (R between 0 and 0.25, left panel), moderate (R between 0.25 and 0.75, middle panel) and large (R above 0.75, right panel) contribution. Dashed lines: correlation thresholds for identification of an interaction (R < -0.75 or R > 0.75). **B)** Impact of confounding metabolites on the identification of promoter-metabolite interactions. For each simulated promoter and metabolite (n = 10000), an additional set of metabolites with 25% (blue), 50% (red), 75% (orange), or 95% (purple) similarity to the original data (similarity defined as Pearson correlation coefficient between original and additional metabolite data) was generated and used to calculate the correlation between log-transformed promoter activity (after removing expression machinery contribution) and each confounding metabolite (corresponding to a false-positive regulatory signal). False discovery and true positive rates were calculated using the p-value of the correlation as a metric. Horizontal dashed line: true positive rate = 0.75, corresponding to a correlation coefficient threshold of 0.74 (with p-value 0.000047). Left vertical dashed line: FDR when assuming a true positive rate of 0.75 and a similarity of confounding metabolites of 0.75. Right vertical dashed line: FDR when assuming a true positive rate of 0.75 and a similarity of confounding metabolites of 0.95.

3. Algorithm to systematically identify regulatory metabolites

Perform separately for each promoter:

1. Subtract first singular vector to obtain the isolated specific regulation S across conditions
2. Linear regression of equation S = p * M, where p denotes the promoter- and metabolite specific parameter (corresponding to the lumped parameter ($\alpha_{i,l} * \beta_{l,k}$) in equation 3 in the main text) to be determined in the regression. Exclude conditions in which the metabolite could not be quantified
3. Calculate Pearson correlation coefficient R between S and its reconstruction based on p * M
4. For each metabolite pair M1 + M2: linear regression of equation S = p1 * M1 + p2 * M2 as above. Exclude conditions in which either of the metabolites could not be quantified.
5. Calculate Pearson correlation coefficient R between S and its reconstruction based on (p1 * M1 + p2 * M2)
6. Assess whether any metabolite pair explains S better than the best single metabolite based in the difference in Akaike Information Criterion (AIC):
    a. Calculate AIC and ΔAIC as described in material and methods for each metabolite pair as well as the best single metabolite.
    b. Identify metabolite pairs which pass all cut-offs: R > 0.75; at least 20% increase in R compared to the best single metabolite; ΔAIC above a cut-off corresponding to a relative likelihood of > 50 (Burnham *et al*, 2011).
7. If more than one metabolite pair pass all aforementioned cut-offs: select the one with the highest correlation coefficient as the potential regulatory metabolite pair of the respective promoter
8. If no metabolite pair passes all cut-offs: Select the single metabolite with the highest correlation coefficient (given a general cut-off of R > 0.75) as the potential single regulatory metabolite of the respective promoter
9. If no single metabolite passes the correlation coefficient cut-off of R > 0.75: don’t assign any regulatory metabolites to the respective promoter
10. The sign of each metabolite-promoter interaction was determined based on the fitted regression parameters p (or p1, p2): if p < 0, the interaction is defined as negative (metabolite has negative effect on S), if p > 0, the interaction is defined as positive (metabolite has positive effect on S). The sign of the correlation coefficient (e.g. in figure 3B or S13) is then set accordingly.

Appendix figures

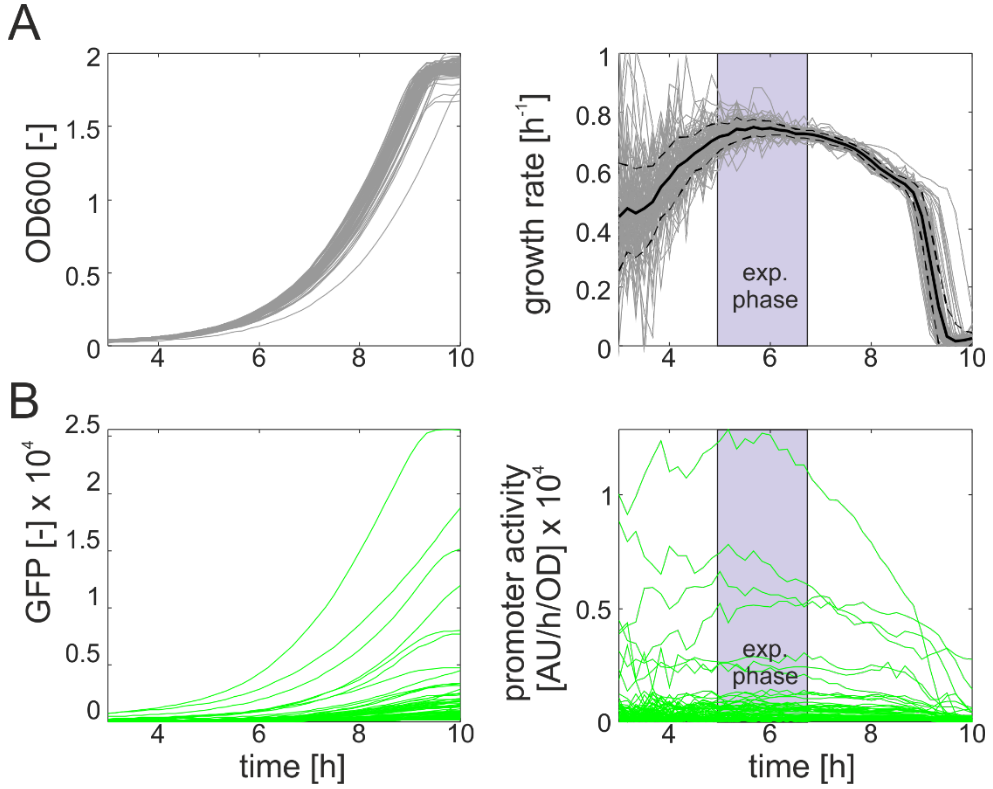

Appendix Figure S1. Quantification of steady state promoter activity and growth rate from OD600 and GFP time course data. Example condition: M9 minimal medium with 2 g/L glucose. A) Left panel: Time course OD600 curves of 96 reporter strains (95 promoter reporter strains, plus one promoter-less reporter strains to determine the background signal). Right panel: Corresponding point-to-point growth rate (calculated by two-point finite difference numerical approximation, see (Gerosa *et al*, 2013)). Black continuous line: mean point-to-point growth rate across all strains. Dashed black lines: corresponding standard deviation. B) Left panel: time course of GFP measurements (see methods). Right panel: Corresponding point-to-point promoter activity (see methods and (Gerosa *et al*, 2013)). Exponential phase was identified visually as the time window with the highest and approximately constant growth rate (marked with violet box), and mean promoter activity and growth rate were calculated in this window for each promoter.

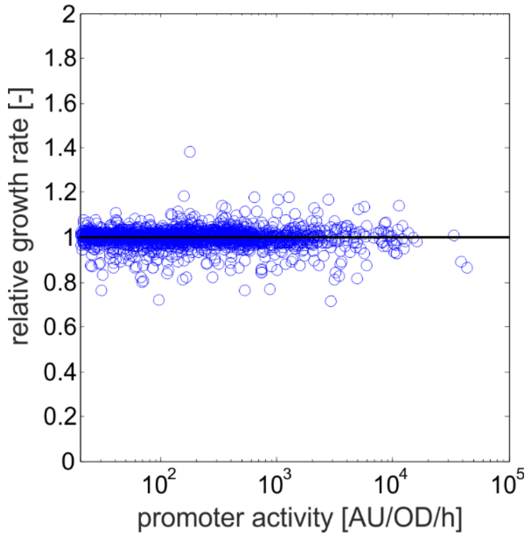

Appendix Figure S2. GFP expression does not impair growth rate. Promoter activities of all 95 tested promoter strains in all 26 tested conditions plotted against the growth rate of the respective strain (relative to the mean growth rate in the same condition).

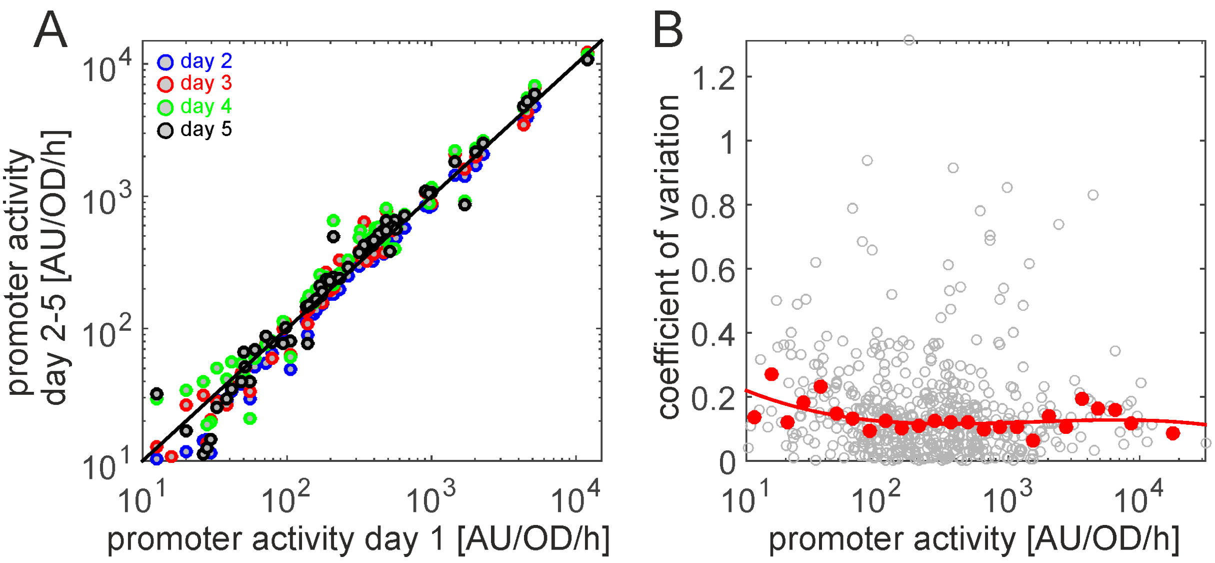

Appendix Figure S3. Day-to-day reproducibility and estimate of coefficient of variation of promoter activity measurements. All 64 promoters with promoter activities above background (see main text) were considered. **A)** Promoter activity during exponential growth (condition: M9 glucose) was determined in five independent experiments. Median day-to-day variation is 15%. **B)** Coefficient of variation (standard deviation divided by mean) in 9 conditions plotted against the mean promoter activity (gray circles, based on 2 to 5 replicates). Red circles: median coefficient of variation for 24 evenly spaced bins (in log scale). Red line: polynomial fit of median coefficients of variation, which allows to estimate the coefficient of variation also for promoters without replicate measurements (Keren *et al*, 2013).

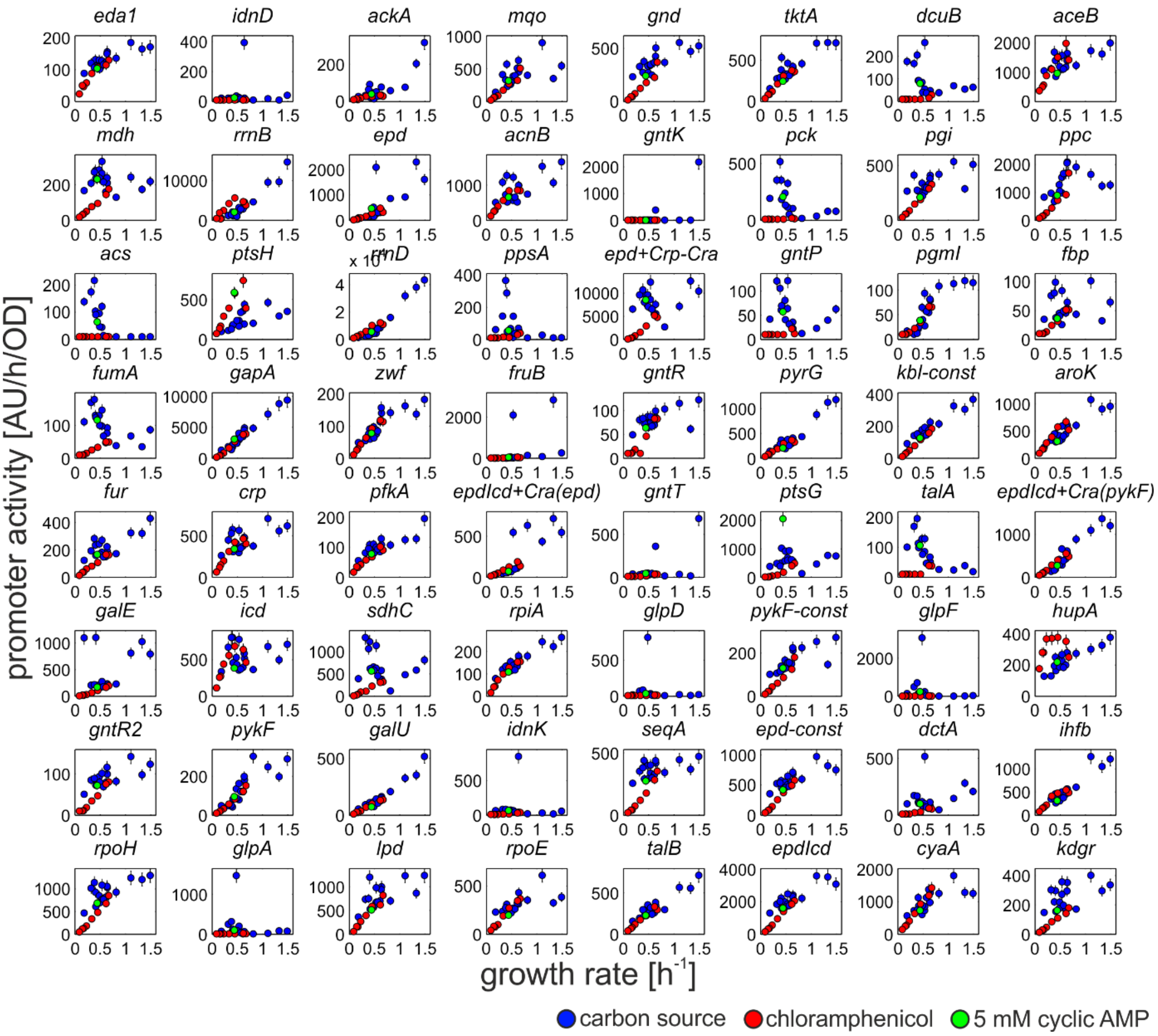

Appendix Figure S4. Steady state promoter activity of 64 promoters in 26 conditions. Error denote standard deviation and were estimated based on day-to-day reproducibility measurements (see appendix figure 3).

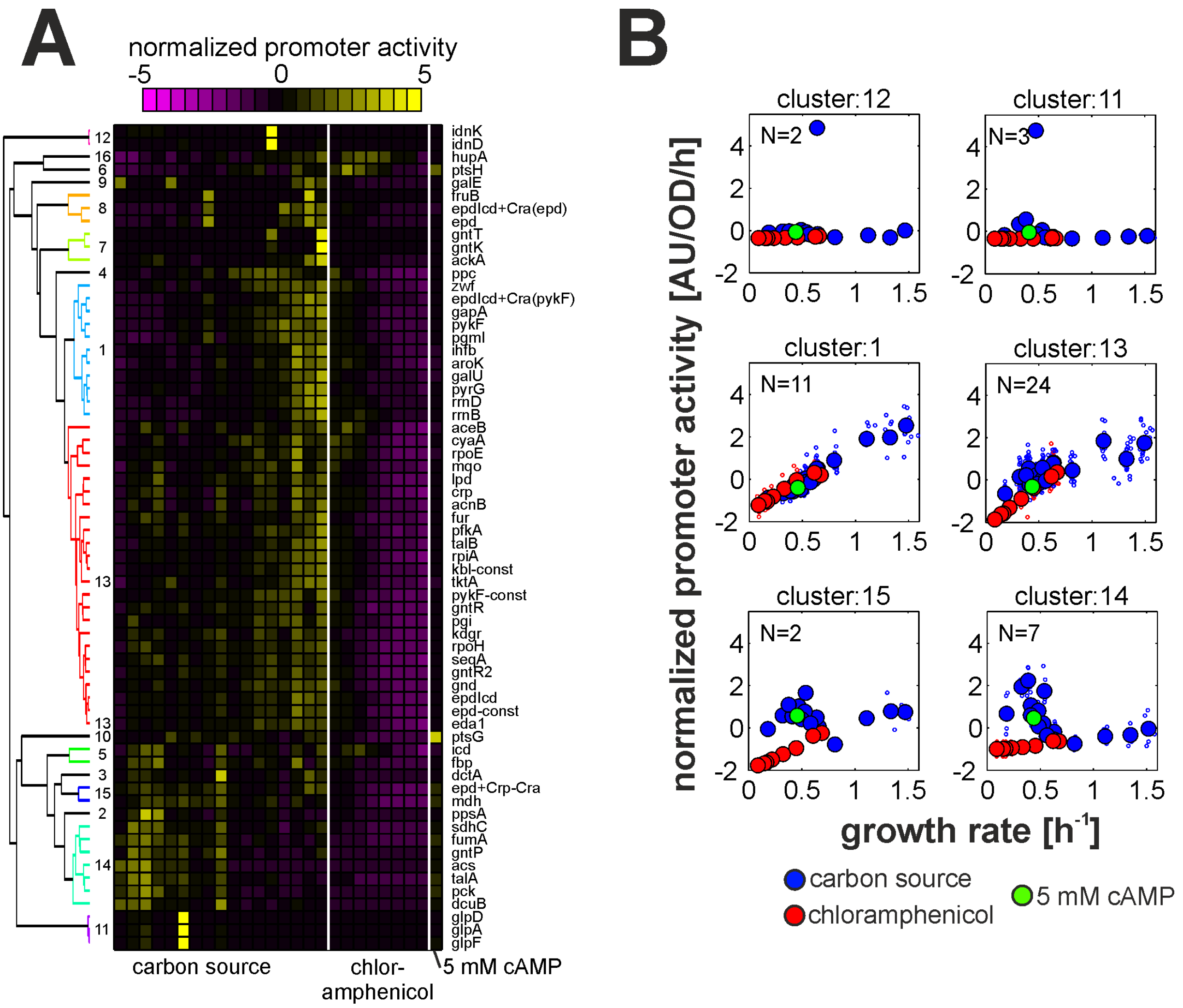

Appendix Figure S5. Hierarchical clustering of central metabolic promoters. A) Steady state promoter activities in various carbon sources as well as different sub-lethal doses of chloramphenicol and supplementation of 5 mM cyclic AMP (same data as in main figure 1). Promoter activities were normalized by z-score normalization and sorted by 1-dimensional hierarchical clustering across promoters. MATLAB commands: 1. step *pdist* (distance metric: Pearson correlation coefficient between promoters), 2. step *linkage* (algorithm: unweighted average distance), 3. step *cluster* (cut-off: 0.225, clustering metric: distance). Carbon sources were sorted by increasing growth rate (from left to right), Chloramphenicol data were sorted by increasing chloramphenicol concentrations (from left to right). Last column: M9 glucose with 5 mM cyclic AMP (cAMP). **B)** Selected clusters for each conditions plotted against their respective steady state growth rate. Large filled circles: mean promoter activities of all promoters in respective cluster. Small non-filled circles: promoter activities of all individual promoters in respective cluster.

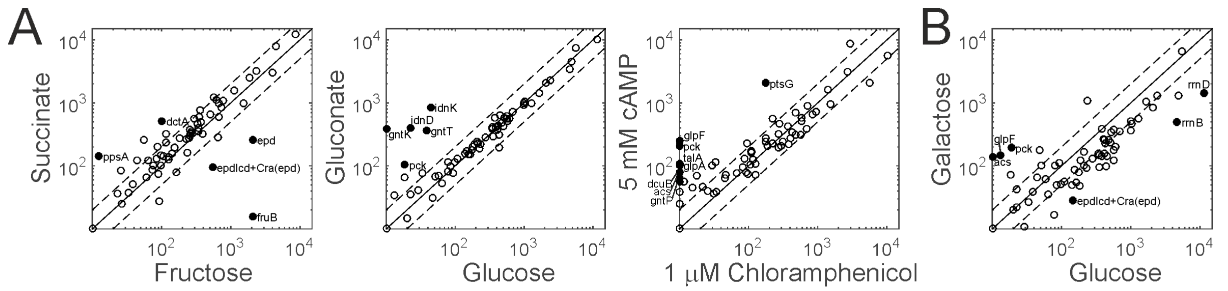

Appendix Figure S6. Measured steady state promoter activity in pairwise conditions. A) Condition pairs with highly similar growth rates. Fructose: 0.53 h^{-1}. Succinate: 0.53 h^{-1}. Glucose: 0.64 h^{-1}. Gluconate: 0.65 h^{-1}. 1 μM Chloramphenicol in M9 glucose: 0.45 h^{-1}. 5 mM cAMP in M9 glucose: 0.44 h^{-1}. **B) Pair of conditions which differ greatly in growth rate**. Glucose: 0.64 h^{-1}. Galactose: 0.18 h^{-1}. Median absolute log2-fold change between Glucose and Galactose conditions: 1.1. Black circles: promoters whose activity deviates by more than 5-fold in the pairwise conditions (>5x, or <0.2x), labeled with the respective promoter name. Dashed lines: commonly used log2 fold-change cut-offs (log2 fold-change > 1, or < -1).

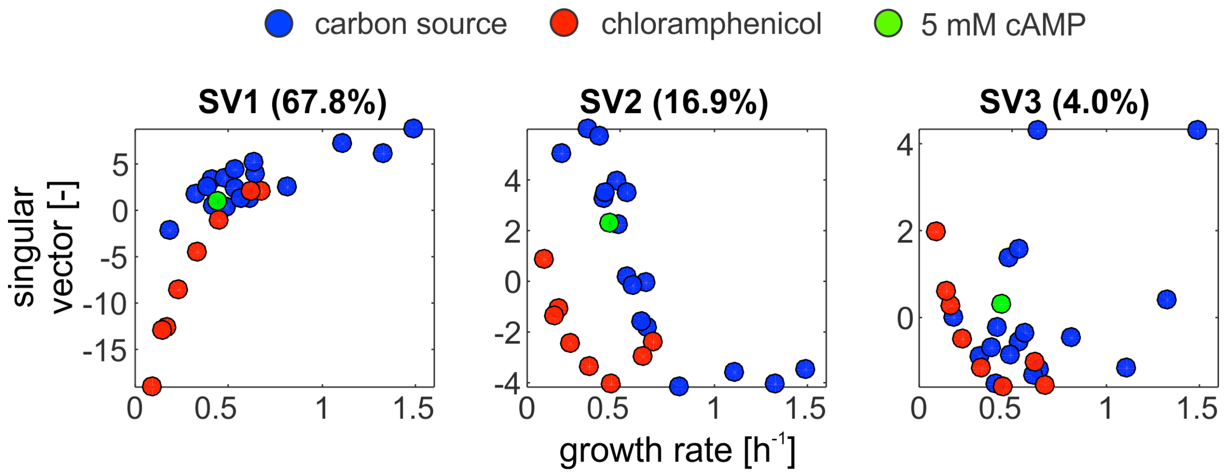

Appendix Figure S7. Singular value decomposition of steady state promoter activity. Shown: Top three singular vectors explaining most of the data set variability. Singular value decomposition was performed on log transformed and z-score normalized promoter activity data for 64 central metabolic promoters as described in the main text, using the MATLAB command *svds*. Numbers in brackets: percentage of variance in data set explained by respective singular vector.

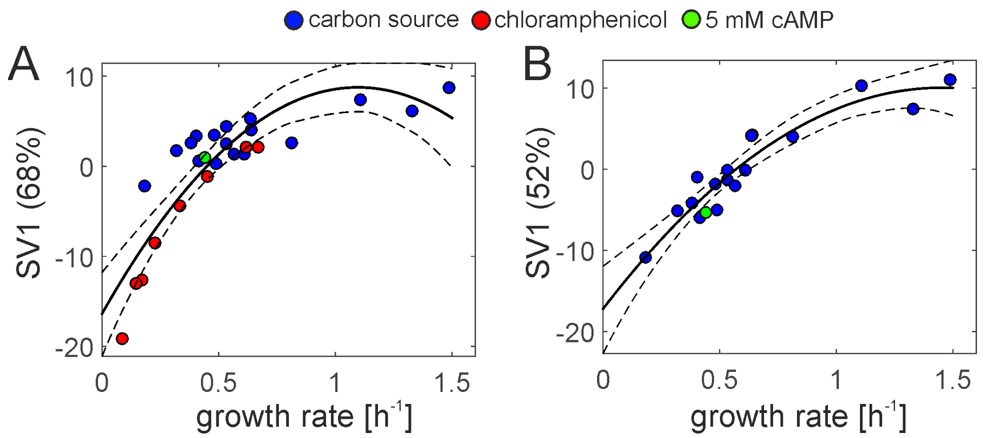

Appendix Figure S8. Singular value decomposition of all tested conditions (A), and excluding chloramphenicol treatment (B). Shown is the first singular vector, which explains 68% (A) and 52% (B) of the variance in the data set, respectively. Black line denotes the polynomial fit of the relationship between SV1 and the growth rate, and the dashed lines denote the 95% confidence interval of the fit.

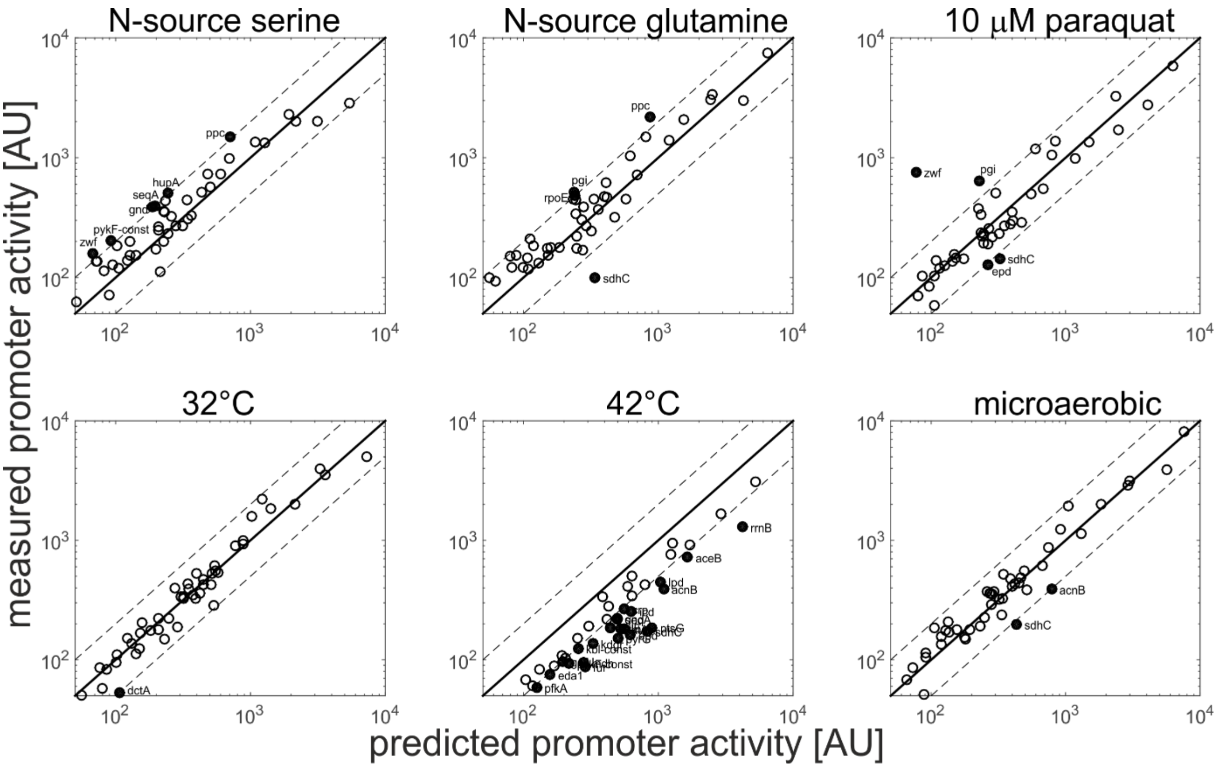

Appendix Figure S9. Measured versus predicted steady state promoter activity of central metabolic promoters in 6 non-carbon source perturbations. Promoter activities were predicted from the steady state growth rate using the relationship between the first singular vector and growth rate depicted in main figure 2B. Filled circles denote promoters whose measured activity deviates more than 2-fold from the prediction (see dashed lines). Microaerobic cultivation was mimicked by incubating the 96-well plate without shaking (barring brief and mild orbital shaking every 6 minutes immediately before OD and GFP measurements to prevent bacterial sedimentation). Note that the two promoters whose activity deviates the most from the growth-dependent prediction upon treatment with the oxidative stress inducing agent paraquat are *zwf* and *pgi*, both of which are activated by the oxidative stress regulator SoxS (Salgado *et al*, 2013).

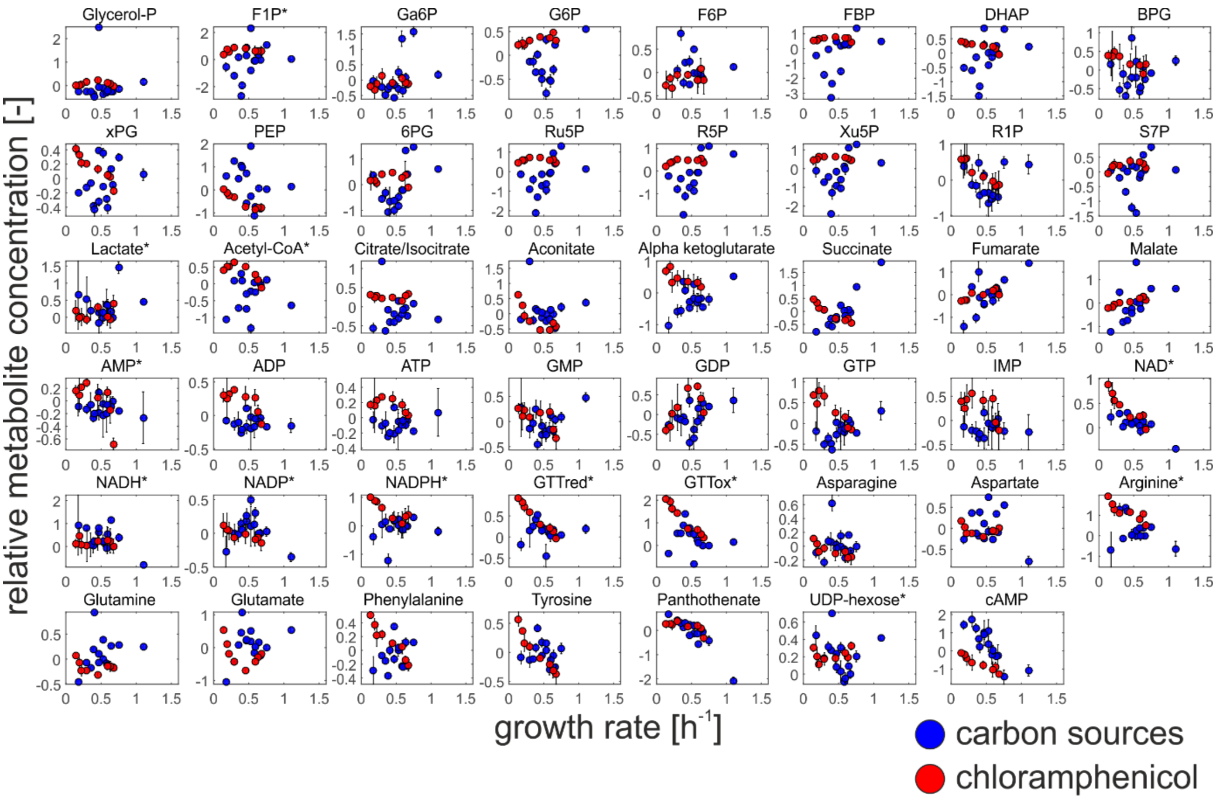

Appendix Figure S10. Intracellular concentrations of central carbon metabolites during exponential growth. Absolute metabolite concentrations of 47 central carbon metabolism metabolites in 23 conditions (transformed using the natural logarithm and normalized by mean metabolite concentration across conditions) as quantified by targeted LC/LC mass spectrometry. Metabolites which were present in the medium were omitted from the quantification. For metabolites marked with (*), no absolute quantification was available. Their concentration was quantified relative to M9 glucose, and then transformed using the natural logarithm. Error bars denote standard deviation of four biological replicates.

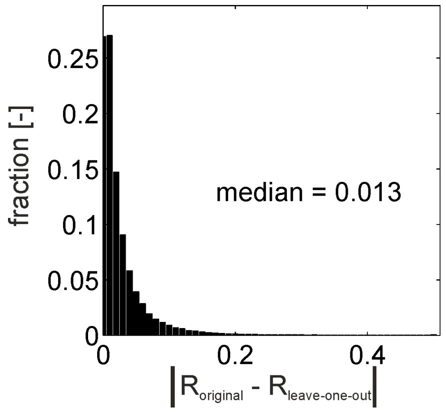

Appendix Figure S11. Robustness of fit between each promoter's specific regulation component and each metabolite against data point elimination. Robustness of fit was performed by systematically omitting one condition and repeating the analysis outlined in figure 3A. Shown here: distribution of absolute difference in correlation coefficient R (Pearson correlation) between the original coefficients (all conditions included) and coefficients when excluding one condition for all combinations of promoters, metabolites, and omitted conditions.

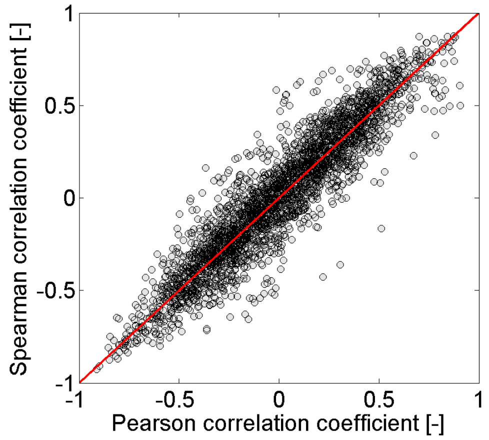

Appendix Figure S12. Comparison of linear (Pearson) and rank (Spearman) correlation as metrics to identify promoter-metabolite interactions. The ability of each metabolite to explain each promoter's specific transcriptional regulation component (see figure 3A) was assessed using Pearson and Spearman correlation coefficients, respectively.

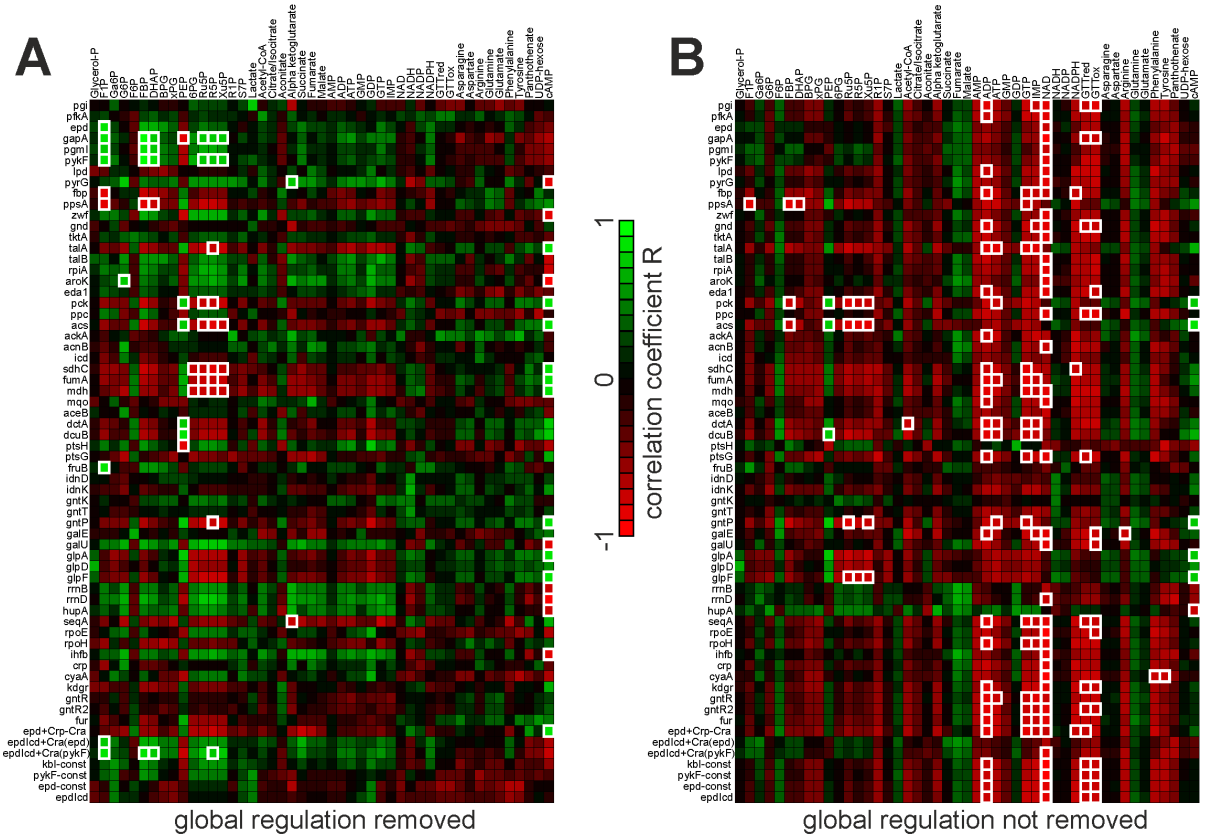

Appendix Figure S13. Identification of promoter-metabolite interactions in absence or presence of global transcriptional regulation. A) Heatmap of Pearson correlation coefficients between measured specific transcriptional regulation (after removal of the respective global regulation component) and its reconstruction based on one metabolite (as described in figure 3A) for all promoter-metabolite pairs. Pairs with correlation coefficients above 0.75 or below -0.75 are shown with thick white edges, all of which were highly significant even after adjusting p-values for multiple hypothesis testing (q-value < 0.001, correction for multiple hypothesis testing as described by (Storey, 2002)). Corresponding data: EV table 7. **B)** Heatmap of Pearson correlation coefficients between log

normalized promoter activity (consisting of global and specific transcriptional regulation) and its reconstruction based on one metabolite (as described in figure 3A) for all promoter-metabolite pairs. Pairs with correlation coefficients above 0.75 or below -0.75 are shown with thick white edges.

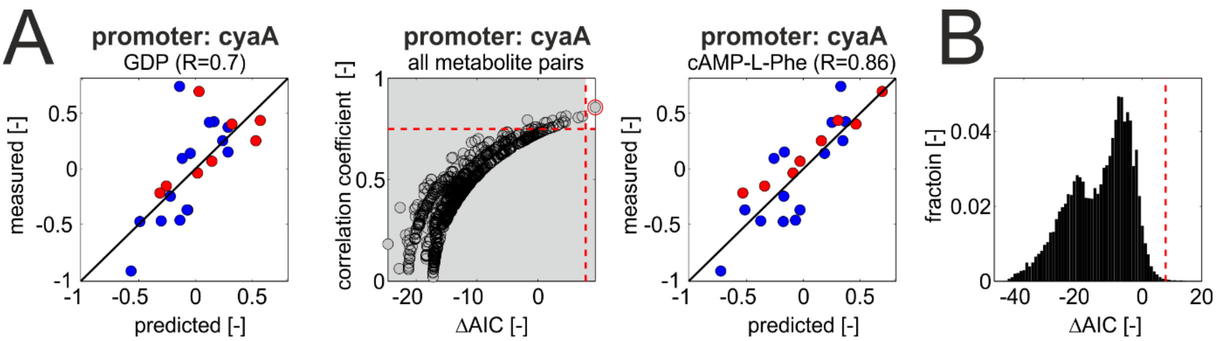

Appendix Figure S14. Identification of pairwise metabolic regulatory signals affecting the specific transcriptional regulation of promoters. A) Outline of approach with example promoter *cyaA*. Left panel: Predicted and measured specific regulation for best single metabolite (GDP), with the correlation coefficient R in brackets. Blue circles: carbon sources. Red circles: chloramphenicol conditions. Middle panel: predictive power of all pairwise metabolite combinations (calculated as the correlation coefficient R between the promoter's measured specific regulation and its prediction based on the respective metabolite pair) plotted against the difference in Akaike Information Criterion (AIC), which penalizes the number of parameters in different models, compared to the best single metabolite ($\Delta AIC = AIC_{bestSingle} - AIC_{metabolitePair}$). Horizontal red dashed line: threshold for R (0.75). Vertical red dashed line: threshold for ΔAIC (based on a relative likelihood threshold of 50, signifying that the respective metabolite pair is 50-times more likely to explain the data than the best single metabolite (Burnham *et al*, 2011)). Right panel: Predicted and measured specific regulation for best metabolite pair, namely cyclic AMP (cAMP) and L-phenylalanine (L-Phe), with the correlation coefficient R in brackets. Blue circles: carbon sources. Red circles: chloramphenicol conditions. **B)** Distribution of ΔAIC across of promoter-metabolite pair combinations. Note that the vast majority of these combinations have negative ΔAIC's, signifying that these metabolite pairs explain the respective promoter's specific regulation worse than the best single metabolite. Vertical red dashed line: threshold for ΔAIC (same as in A).

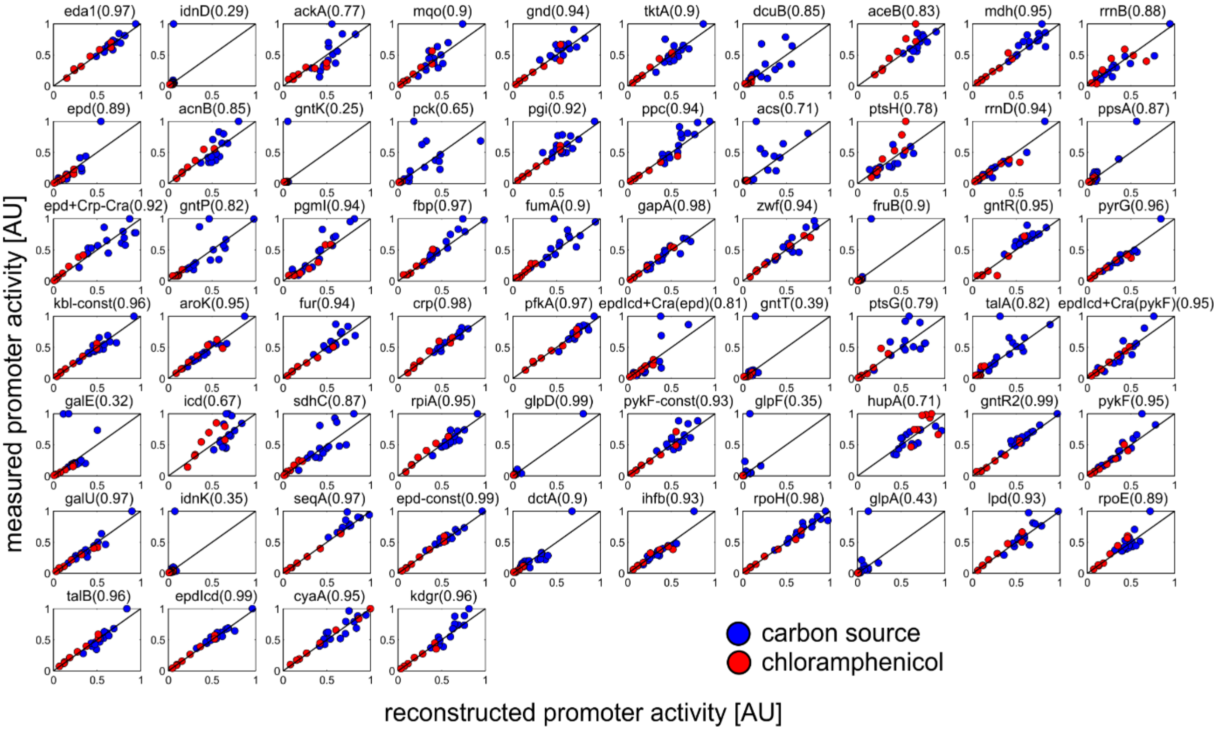

Appendix Figure S15. Reconstruction of individual promoters in 23 conditions based on the quantified regulatory network. The activity of each promoter was reconstructed from its global and specific regulation components based on the network depicted in figure 4A, and then reverted to linear scale. Only conditions with available metabolite and promoter activity data were considered. To aid readability, measured and predicted promoter activities were further normalized to the maximal measured promoter activity of each promoter. In brackets: Pearson correlation coefficient between measured and reconstructed promoter activity.

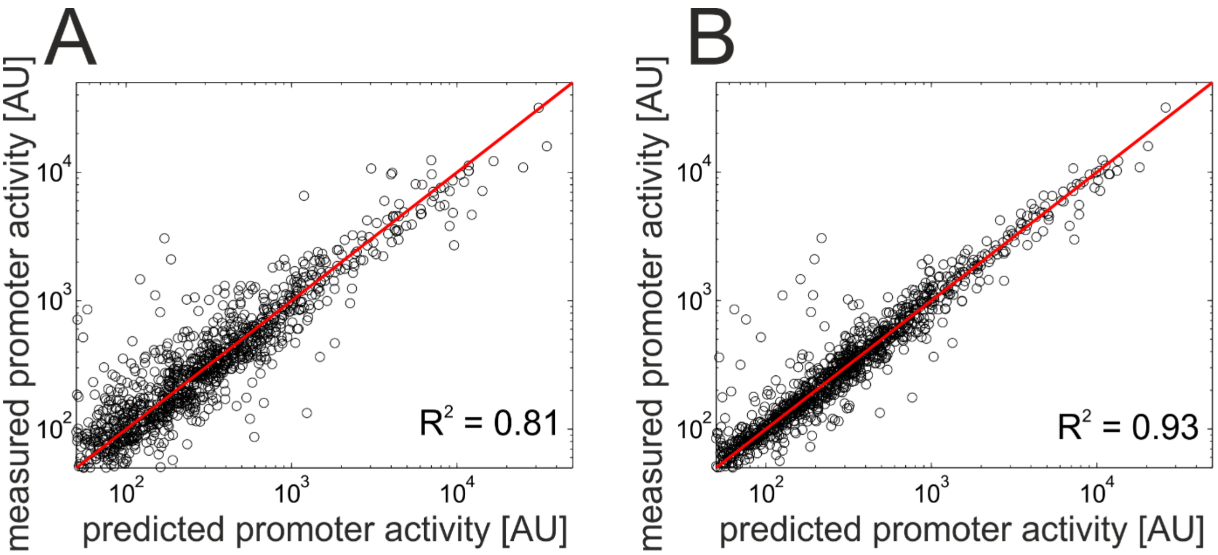

Appendix Figure S16. Leave-one-condition-out cross-validation. A) Comparison of measured and predicted promoter activity for individual conditions that were excluded in the analysis. The impact of global regulation in the excluded condition was predicted from its growth rate based on fitted relationship between growth rate and singular vector 1 depicted in figure 2B. To predict the impact of specific regulation, the topology of the network shown in figure 4A was used as a basis, and the parameters of each promoter-metabolite interaction were re-fitted while omitting the data-point belonging to the excluded condition. Using these re-fitted parameters, each promoter's specific regulation component was then predicted based on the respective metabolite concentration. Finally, each promoter's summed contribution of global and specific regulation was reverted back to linear scale. This procedure was repeated for each condition. R^2 denotes the overall goodness of fit between measured and predicted promoter activity across all conditions and promoters. **B)** Same as A). However, while each promoter's specific regulation component was still predicted from the respective metabolite as described above, its global regulation component was not fitted, but rather directly obtained from singular value decomposition of the experimental data (corresponding to singular vector 1). The improved goodness-of-fit compared to A) suggests that discrepancies between measured in predicted promoter activity in A) are largely caused by deviations of directly quantified global regulation from its growth-rate dependent fit (figure 2B).

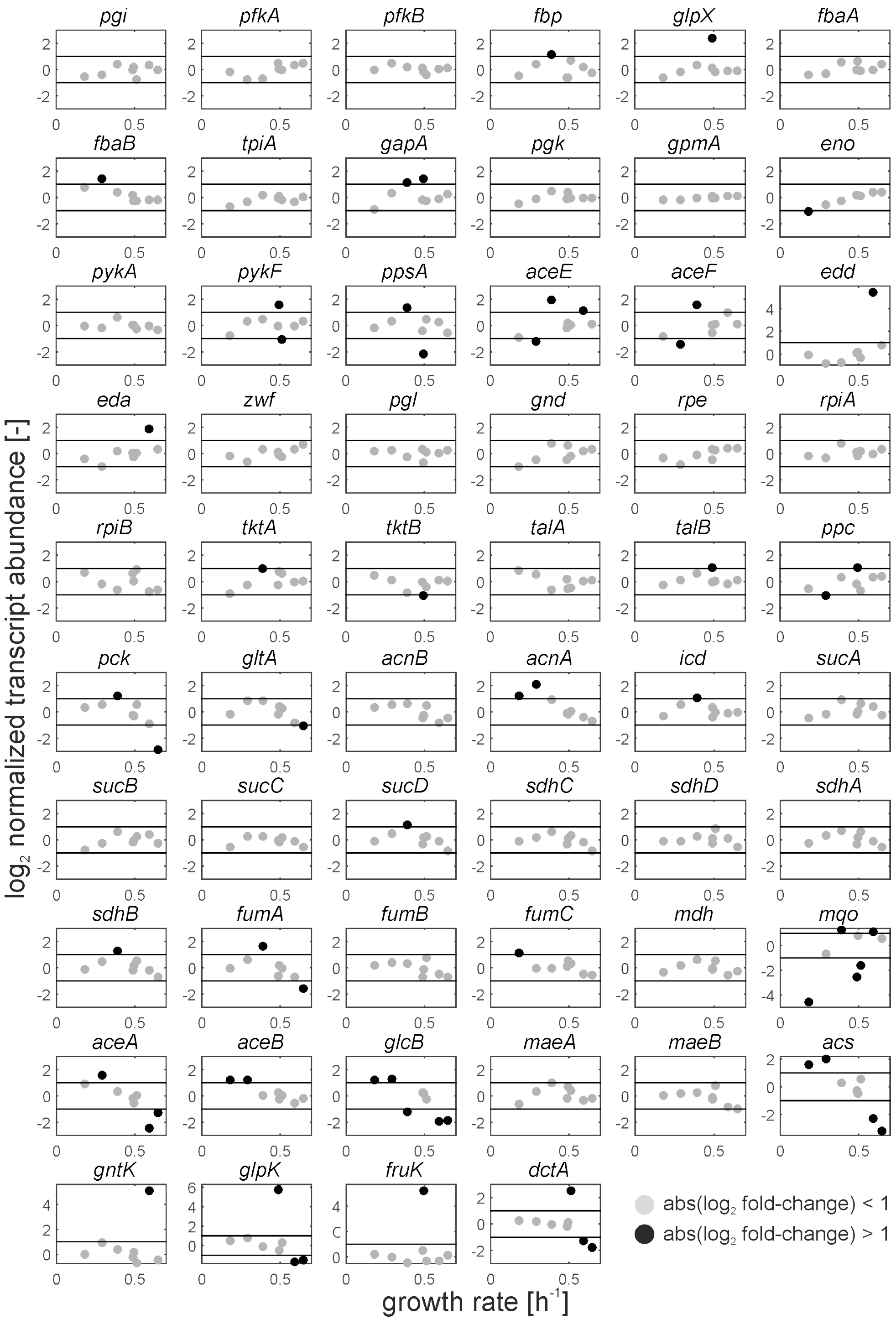

Appendix Supplementary figure S17. Transcript abundance of central metabolic genes in 8 different carbon source conditions. Data from Gerosa et al, 2015. Cell Systems (mean of three biological replicates, log_2 normalized). Grey circles: absolute log_2 fold-change < 1. Black circles: absolute log_2 fold-change > 1.

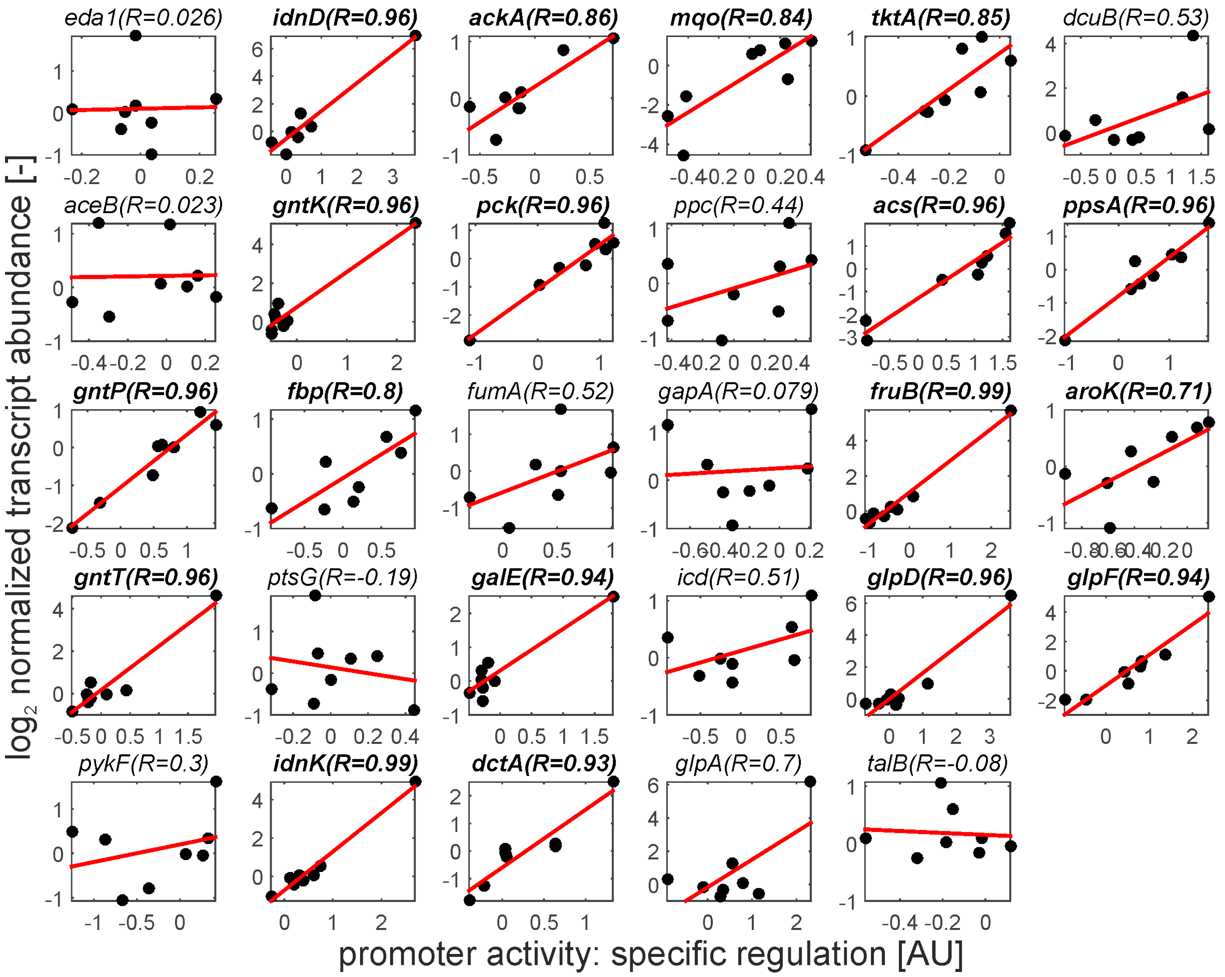

Appendix figure S18. Relationship between transcript abundance and specific regulation component of promoter activity in central metabolic genes. Y-axis: Transcript abundance data as described in appendix figure S17. Only transcripts whose abundance changed >2-fold in at least one condition were considered. X-axis: specific regulation component of corresponding promoter activity data (after normalization and removal of global regulation component, see main text). In brackets: Pearson correlation coefficient between transcript and promoter activity data (**name in bold**: p-value of correlation < 0.05). Red lines: linear regression as visual aid.

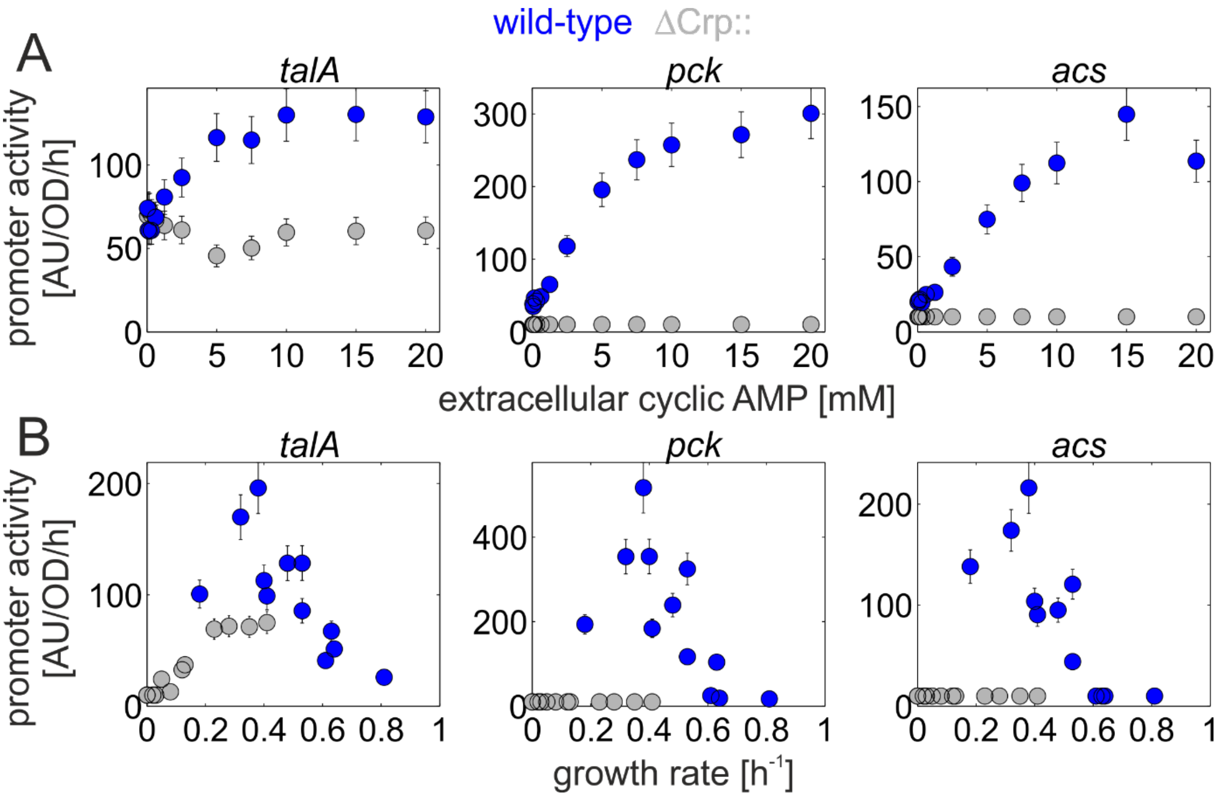

Appendix Figure S19. Promoters *talA* and *pck* are activated by Crp. A) Promoter activity of *talA*, *pck*, and *acs*, during exponential growth in M9 glucose 2g/L with varying concentrations of externally supplemented cyclic AMP, which activates the transcription factor Crp. Blue: wild-type strain, grey: Crp deletion strain. Promoter activity error bars were estimated based on day-to-day reproducibility measurements as described in appendix figure S3. **B)** Promoter activity of *talA*, *pck*, and *acs*, during exponential growth on M9 minimal medium supplemented with 12 different single carbon sources (galactose, acetate, pyruvate, succinate, mannose, glycerol, fructose, gluconate, glucose, lactate, glcNAc, G6P). Blue: wild-type strain, grey: Crp deletion strain. Note that the Crp deletion strain shows impaired growth in all conditions. Promoter activity error bars were estimated based on day-to-day reproducibility measurements as described in appendix figure S3. Data are available in EV table 3 – 5 and EV table 3 - 6.

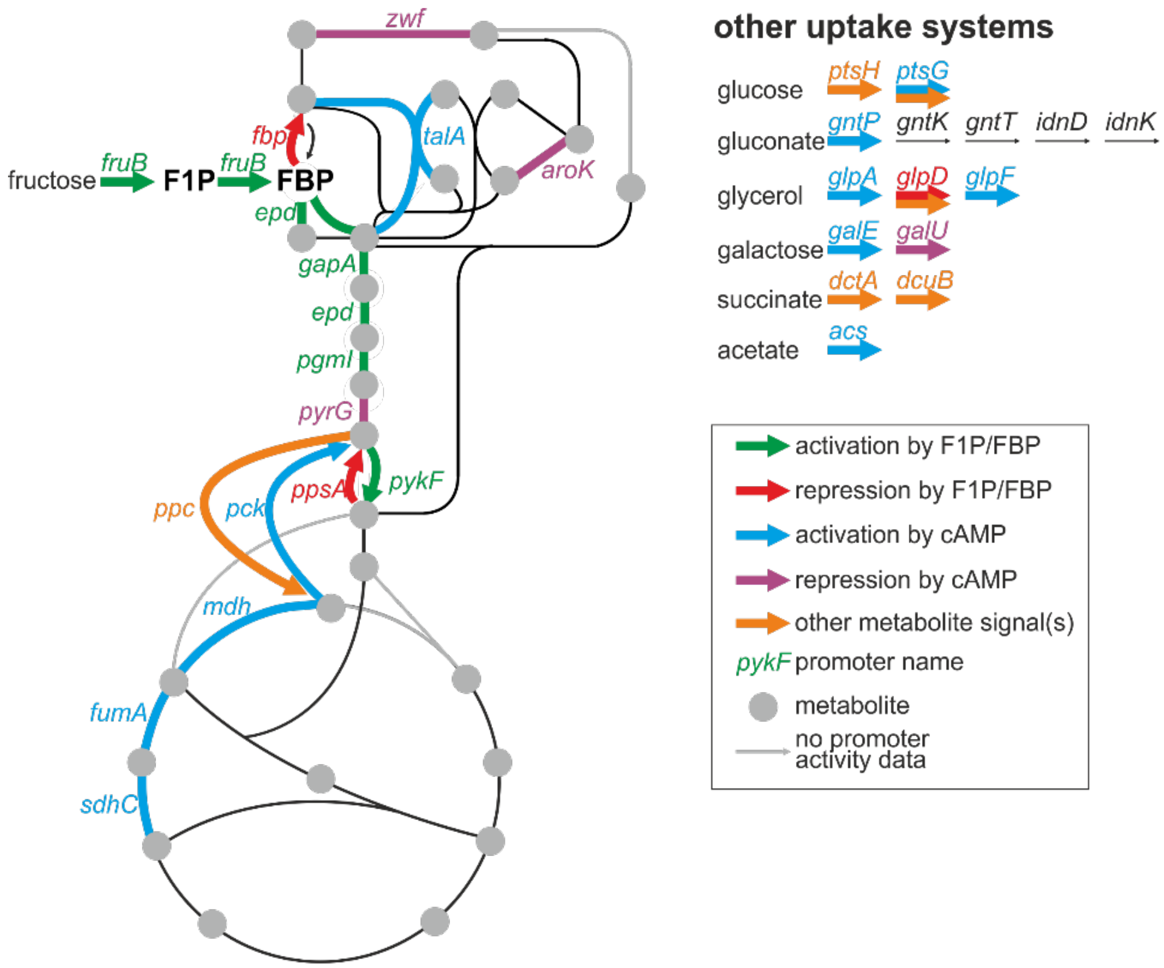

Appendix Figure S20. Schematic of central carbon metabolism (CCM) with corresponding transcriptional metabolite signals. Metabolite signals as depicted in main figure 4A. Each CCM reaction, for which at least promoter was quantified, is colored according to the identified metabolite signal (see accompanying legend). The respective promoter name is written next to the reaction. Note that some promoters, such as *epd*, drive the expression of more than one gene. Information on transcriptional units was obtained from (Salgado *et al*, 2013).

EV tables

EV Table 1: List of promoters used in this study.

EV Table 2: List of conditions used in this study.

EV Table 3: Steady state promoter activity.

EV Table 4: Steady state global regulation as inferred by singular value decomposition.

EV Table 5: Dynamic promoter activities during diauxic shift from glucose to succinate with corresponding prediction based on growth dependent global regulation alone.

EV Table 6: Intracellular metabolite concentrations (relative and absolute concentration).

EV Table 7: Inferred promoter-metabolite and transcription factor-metabolite interaction network.

EV Table 8: Reported transcriptional regulatory network of promoters used in this study.

References

Burnham KP, Anderson DR & Huyvaert KP (2011) AIC model selection and multimodel inference in behavioral ecology: some background, observations, and comparisons. *Behav. Ecol. Sociobiol.* **65:** 23–35

Gerosa L, Haverkorn Van Rijsewijk BRB, Christodoulou D, Kochanowski K, Schmidt TSB, Noor E, Sauer U, Schmidt S, Noor E & Sauer U (2015) Pseudo-transition analysis identifies the key regulators of dynamic metabolic adaptations from steady-state data. *Cell Syst.* **1:** 270–282

Gerosa L, Kochanowski K, Heinemann M & Sauer U (2013) Dissecting specific and global transcriptional regulation of bacterial gene expression. *Mol. Syst. Biol.* **9:** 658

Ishihama A, Kori A, Koshio E, Yamada K, Maeda H, Shimada T, Makinoshima H, Iwata A & Fujitac N (2014) Intracellular concentrations of 65 species of transcription factors with known regulatory functions in Escherichia coli. *J. Bacteriol.* **196:** 2718–2727

Keren L, Zackay O, Lotan-Pompan M, Barenholz U, Dekel E, Sasson V, Aidelberg G, Bren A, Zeevi D, Weinberger A, Alon U, Milo R & Segal E (2013) Promoters maintain their relative activity levels under different growth conditions. *Mol. Syst. Biol.* **9:** 701

Salgado H, Peralta-Gil M, Gama-Castro S, Santos-Zavaleta A, Muñiz-Rascado L, García-Sotelo JS, Weiss V, Solano-Lira H, Martínez-Flores I, Medina-Rivera A, Salgado-Osorio G, Alquicira-Hernández S, Alquicira-Hernández K, López-Fuentes A, Porrón-Sotelo L, Huerta AM, Bonavides-Martínez C, Balderas-Martínez YI, Pannier L, Olvera M, et al (2013) RegulonDB v8.0: omics data sets, evolutionary conservation, regulatory phrases, cross-validated gold standards and more. *Nucleic Acids Res.* **41:** D203-13

Storey JD (2002) A direct approach to false discovery rates. *J. R. Stat. Soc. Ser. B (Statistical Methodol.* **64:** 479–498
